# Complementary and Integrated Studies of Longevity and Healthy Aging

**DOI:** 10.1093/geroni/igaa057.3126

**Published:** 2020-12-16

**Authors:** Steven Cummings, Thomas Perls, Evan Hadley

## Abstract

Five NIH-funded studies, the Long Life Family Study (LLFS, U19), the Longevity Consortium (LC, U19), Longevity Genomics (U24), and Protective Omics Profiles in Centenarians (UH2) work together to triangulate on mechanisms of extreme longevity and healthy aging with the ultimate goal of discovering predictors and targetable pathways. Linkage analyses by LLFS identified extremely strong genetic linkage peaks for cross-sectional as well as longitudinal trajectory rates-of-change phenotypes. Deep sequencing suggests these peaks are driven by rare, protective variants in selected pedigrees. In cross-species studies (UH2, LC), genomics, metabolomics and proteomics are used to exploit many-fold variances in natural life spans to discover protective mechanisms that explain some of these differences. Proteome analysis reveal several longevity-related proteins such as Cip1/p21, FOXO3, TOP2A, AKT1, RICTOR, INSR and SIRT6 harboring post translational modification sites that preferentially appear in short- versus long-lived species. The U24 effort developed a tool using genetically-mediated gene expression to prioritize genes for longevity translational efforts. We found that BLOC1S1 was associated with longevity and protection from atrial fibrillation and hearing loss without being associated with adverse events. This novel target is undergoing functional characterization. A proteomic assay (4,131 proteins, Somascan) annotated by genome-wide association study results in a total of 1,797 centenarians and 3,685 controls divided into independent discovery and replication sets, discovered significant and replicated over-expression (thus, pro-longevity) of BIRC2 and under-expression of APOB in carriers of the APOE ɛ-2 allele. A novel protein signature of rs2184061 (CDKN2a/CDKN2B in chromosome 9) was also associated with slower aging.

